# Characterization of a Primate Blood-Brain Barrier Co-Culture Model Prepared from Primary Brain Endothelial Cells, Pericytes and Astrocytes

**DOI:** 10.3390/pharmaceutics13091484

**Published:** 2021-09-16

**Authors:** Daisuke Watanabe, Shinsuke Nakagawa, Yoichi Morofuji, Andrea E. Tóth, Monika Vastag, Jun Aruga, Masami Niwa, Mária A. Deli

**Affiliations:** 1 Department of Medical Pharmacology, Nagasaki University Graduate School of Biomedical Sciences, 1-12-4 Sakamoto, Nagasaki 852-8523, Japan; watanabe@pharmacocell.co.jp (D.W.); aruga@nagasaki-u.ac.jp (J.A.); 2 BBB Laboratory, PharmaCo-Cell Co., Ltd., Nagasaki 852-8135, Japan; niwa@pharmacocell.co.jp; 3 Department of Pharmaceutical Care and Health Sciences, Faculty of Pharmaceutical Sciences, Fukuoka University, 8-19-1 Nanakuma, Jonan-ku, Fukuoka 814-0180, Japan; shin3@fukuoka-u.ac.jp; 4 Department of Neurosurgery, Nagasaki University Graduate School of Biomedical Sciences, 1-7-1 Sakamoto, Nagasaki 852-8501, Japan; morofujiyoichi@gmail.com; 5 Institute of Biophysics, Biological Research Centre, Temesvári krt. 62, H-6726 Szeged, Hungary; toth@biomed.au.dk; 6 In Vitro Metabolism Research, Division of Pharmacology and Drug Safety Research, Gedeon Richter Plc., Gyömrői út 19-21, H-1103 Budapest, Hungary; m.vastag@richter.hu

**Keywords:** blood-brain barrier, brain endothelial cell, astrocyte, pericyte, co-culture, transporter, transendothelial electrical resistance, drug permeability

## Abstract

Culture models of the blood-brain barrier (BBB) are important research tools. Their role in the preclinical phase of drug development to estimate the permeability for potential neuropharmaceuticals is especially relevant. Since species differences in BBB transport systems exist, primate models are considered as predictive for drug transport to brain in humans. Based on our previous expertise we have developed and characterized a non-human primate co-culture BBB model using primary cultures of monkey brain endothelial cells, rat brain pericytes, and rat astrocytes. Monkey brain endothelial cells in the presence of both pericytes and astrocytes (EPA model) expressed enhanced barrier properties and increased levels of tight junction proteins occludin, claudin-5, and ZO-1. Co-culture conditions also elevated the expression of key BBB influx and efflux transporters, including glucose transporter-1, MFSD2A, ABCB1, and ABCG2. The correlation between the endothelial permeability coefficients of 10 well known drugs was higher (R^2^ = 0.8788) when the monkey and rat BBB culture models were compared than when the monkey culture model was compared to mouse in vivo data (R^2^ = 0.6619), hinting at transporter differences. The applicability of the new non-human primate model in drug discovery has been proven in several studies.

## 1. Introduction

Cell-culture based models have greatly contributed to our knowledge on the physiology, pathology, and pharmacology of the blood-brain barrier (BBB) [1,2,3]. In the preclinical phase of drug development, it is key to estimate the brain penetration of potential neuropharmaceuticals, and in this process culture models of the BBB are essential tools [2,4,5,6]. The specific features of cerebral capillary endothelial cells, which form the anatomical basis of the BBB, are organ specific. The dynamic interactions between brain endothelial cells and the neighboring astrocytes and pericytes promote the development and maintenance of BBB functions [7,8]. It is therefore crucial to mimic this cellular complexity and the crosstalk between these three cell types in culture models of the BBB. The first co-culture models, developed in the end of 1980s, were based on cerebral endothelial cells grown in the presence of astrocytes. These well characterized models are still used for drug permeability and discovery studies [1,2,3]. We have demonstrated that not only astrocytes, but brain pericytes can also increase the tightness of the paracellular barrier of rat brain endothelial cells in culture [9]. Based on this observation we have pioneered a triple co-culture model of the BBB using primary rat brain endothelial cells, pericytes, and astrocytes [10]. The number of triple co-culture BBB models from different species and cell sources, including primary cells, cell lines, and stem-cell derived cells, has been expanded in the last decade [3].

While the basic defense mechanisms and functions of the BBB are well conserved in mammals, species differences have been described in BBB transport systems. Using quantitative proteomics, the expression of active efflux and influx transporters in human brain capillaries were close to those from monkey, but more different as compared to mouse brain capillaries [11,12], indicating the limitations of the widely used rodent models. While the best models for the prediction of brain penetration of pharmaceutics in patients would be human cell-based systems, the current human BBB models have several weak points. The expression of tight junction proteins and the paracellular barrier of human brain endothelial cell lines is not tight enough for testing small molecules [3,6]. The paracellular tightness of the stem cell based human BBB models is much stronger, and high electrical resistance values were described [3], but some of these models do not show a typical endothelial cytoarchitecture [13]. Primary brain endothelial cells have both appropriate barrier tightness and microvascular endothelial characteristics [1,3,5,6,8]. The reproducibility of co-culture BBB models is usually good within laboratories, but the properties of models may differ between different groups [1,3].

Our aim was to develop a new primate co-culture BBB model using primary cultures of monkey brain capillary endothelial cells, rat pericytes and rat astrocytes, characterize its paracellular barrier properties, and demonstrate the presence of selected key BBB transporters. To assess the model’s applicability for drug transport studies we tested the permeability of 10 well-known pharmacons and compared the data with results obtained on a rat triple co-culture model and brain penetration in mice.

## 2. Materials and Methods

### 2.1. Animals

Wistar rats were obtained from Japan SLC Inc. (Shizuoka, Japan). Rats were housed under specific pathogen-free conditions in an air-conditioned room and fed standard laboratory chow ad libitum, in accordance with institutional guidelines. Based on the Guide for the Care and Use of Laboratory Animals from the Ministry of Education, Culture, Sports, Science, and Technology, Japan, all experimental procedures were reviewed by Institutional Animal Care and Use Committee of Nagasaki University and finally approved by the University’s president (license no.: 1805011452, date of approval: 1 May 2018). Brains from cynomolgus monkeys (*Macaca fascicularis*) were purchased and transported from Ina Research Inc. (Nagano, Japan) to the laboratory in cold phosphate-buffered saline (PBS) with Ca^2+^ and Mg^2+^ (Wako Pure Chemical Industries, Ltd., Osaka, Japan) supplemented with amphotericin B solution (2.5 μg/mL) within 24 h. The animals were tested for antibodies to herpes B-virus, *Shigella*, *Salmonella*, and tuberculosis and showed negative results on screening.

### 2.2. Materials and Reagents

All reagents used in the study were purchased from Sigma (St. Louis, MO, USA), unless otherwise indicated.

### 2.3. Primary Cell Cultures

Primary cultures of monkey brain capillary endothelial cells (MBECs) and astrocytes were prepared from 3 to 5-year-old male *Macaca fascicularis* monkeys. The preparation of primary cultures of MBECs were based on our previously described method for rat brain endothelial cells [9,10]. Briefly, meninges and surface vessels were removed, and gray matter was minced into small pieces. The minced gray matter was digested in a mixture of collagenase type 2 (340 U/mL, Worthington, OH, USA), and DNase (15 μg/mL) for 1.5 h at 37 °C. The cell pellet was separated by centrifugation in 20% bovine serum albumin (BSA)-DMEM (1000× *g*, 25 min). The microvessels obtained in the pellet were further digested with collagenase-dispase (1 mg/mL, Roche, Basel, Switzerland) and DNase (6.7 μg/mL) in 15 mL of DMEM for 45 min at 37 °C. Microvessel endothelial cell clusters were separated on a 33% continuous Percoll gradient at 1000× *g* for 10 min. A white color layer, above the red layer consisting of red blood cells near the bottom of the tube, contained the microvessel fragments. The microvessel fragments were collected, washed in DMEM, and plated on plastic dishes coated with collagen type IV and fibronectin (both 0.1 mg/mL). MBEC cultures were maintained in DMEM/F12 supplemented with 10% plasma-derived serum (PDS, Animal Technologies, Tyler, TX, USA), basic fibroblast growth factor (bFGF, Roche, Switzerland, 1.5 ng/mL), heparin (100 μg/mL), insulin (5 μg/mL), transferrin (5 μg/mL), sodium selenite (5 ng/mL) (insulin-transferrin-sodium selenite media supplement), and gentamicin (50 μg/mL). During the first 2 days of culture, the medium of endothelial cells also contained 4 μg/mL puromycin to eliminate P-glycoprotein negative, contaminating cell types [14]. When MBEC cultures were confluent, cells were trypsinized, seeded to culture inserts, and used for experiments at passage 1.

Monkey astrocytes were isolated from a piece of gray matter. The gray matter was mechanically dissociated and digested with papain (1 mg/mL). Dissociated cells were seeded in cell culture flasks coated with poly-L-lysine. Cells were maintained in DMEM supplemented with 10% fetal bovine serum (FBS). After 2 weeks, flasks with confluent cultures were shaken to obtain pure astrocyte culture.

Rat brain endothelial cells (RBECs) were isolated from male Wistar rats (3 to 4 weeks old). RBECs were isolated and cultured using the method described for MBECs. Rat cerebral astrocytes and pericytes were obtained from neonatal rats and 3-week-old male rats, respectively, as we described previously [9,10] (see Supplementary Methods). Both pericytes and astrocytes were cultured in DMEM medium supplemented with 10% FBS and used at passages 2 or 3.

### 2.4. Construction of the BBB Model

To prepare the in vitro co-culture models (EPA), pericytes (2 × 10^4^ cells/cm^2^) were cultured on the bottom sides of collagen-coated culture inserts (Transwell clear, polyester membrane, 0.4 μm pore size, Corning Costar, NY, USA), and astrocytes (0.5 × 10^5^ cell/cm^2^) were seeded on the collagen-coated well of a 24-well culture plate. Cells were let to adhere overnight, and endothelial cells (2 × 10^5^ cells/cm^2^) were seeded on the inside of the inserts (top side of the membranes) and placed in the wells of the 24-well culture plates. An endothelial cell monolayer model (E00) was constructed by a similar method as the co-culture model, except for the procedure of seeding of pericytes and astrocytes. BBB models were maintained in RBEC medium supplemented with 550 nM hydrocortisone [10,14].

### 2.5. Immunohistochemistry

For immunohistochemical characterization, MBECs were stained with anti-claudin-5, occludin, ZO-1 (Invitrogen Corporation, Waltham, MA, USA), or von Willebrand factor antibodies. Astrocytes were stained with anti-GFAP antibody (Progen Scientific Ltd., Mexborough, UK). All primary antibodies were used at a dilution of 1:100. As secondary antibodies Alexa Fluor 488 conjugated donkey anti-rabbit and anti-mouse immunoglobulins (both from Invitrogen Corporation) were used at a dilution of 1:1000. The source and catalogue number of antibodies is listed in Table S1. To counterstain cell nuclei TO-PRO-3 Iodide (Invitrogen Corporation) was used at a dilution of 1:400. Cultured cells were fixed in 3% paraformaldehyde in PBS for 10 min and permeabilized with 0.1% Triton X-100 for 10 min. Cells were blocked with 3% bovine serum albumin and were incubated with primary antibodies overnight at 4 °C. After washing, cells were incubated for 1 h at room temperature with secondary antibodies and TO-PRO-3. Cells were washed three times with PBS, and preparations were mounted with Gel Mount (Biomeda, Foster City, CA, USA) and staining was examined using a Zeiss LSM 5 Pascal Confocal laser scanning microscope (Carl Zeiss AG, Oberkochen, Germany).

### 2.6. Reverse Transcription Polymerase CHAIN Reaction

For analysis of expression of transporters on each BBB model cultured for four days, total RNA was isolated with a RNeasy Mini Kit (Qiagen, Hilden, Germany) according to the manufacturer’s instructions. During RNA purification, genomic DNA was eliminated using RNase-Free DNase Set (Qiagen, Hilden, Germany). Concentration and purity (A260/280) of total RNA was analyzed by NanoDrop 1000 (Thermo Fisher Scientific, Waltham, MA, USA). First strand cDNA was synthesized from 1 μg total RNA with the Reverse Transcription System (Promega, Madison, WI, USA). The generated cDNA was stored at −20 °C until use. Polymerase chain reaction (PCR) fragments for transporters were amplified using the primer pairs shown in Table 1. The primers were designed using Primer-BLAST (NCBI, NIH, Bethesda MD, USA) and synthesized by Sigma-Genosys Ltd. Haverhill, UK. The specificity of each primer pair was verified by the appearance of a single band in agarose gels (Figure 2a) and a single peak in a melting curve (Figure S1). PCR was performed in a final volume of 20 μL containing 0.5 μL of template cDNA, 1.6 μL of dNTP mixture, 2 μL of 10 × PCR buffer, 0.5 unit of Takara Taq™ Hot Start Version polymerase (Takara Bio, Inc., Shiga, Japan), and 1 μM of each primer, using a PCR Express II thermal cycler (Thermo Electron Corp., Waltham, MA, USA). PCR from the first strand cDNAs were performed with Takara Taq™ Hot Start Version (Takara Bio Inc., Shiga, Japan) and PCR Express II thermal cycler (Thermo Electron Corp., Waltham, MA, USA). PCR was performed with 40 cycles of denaturation at 98 °C for 10 s, annealing at 60 °C for 30 s, and extension at 72 °C for 15 s. PCR products were separated by electrophoresis on 1.5% agarose gels and stained with ethidium bromide. Real-time PCR amplifications were performed using the ABI PRISM 7900HT Sequence Detection System (Applied Biosystems, Waltham, MA, USA).

Each PCR was performed by mixing 1 μL cDNA and 5 pmol of each primer (Table 1) with the THUNDERBIRD SYBR qPCR Mix (TOYOBO, Osaka, Japan). PCR reactions were as follows: 95 °C for 1 min, 40 cycles at 95 °C for 15 s, 60 °C for 15 s, and 72 °C for 45 s. The cDNA quantities were measured in critical thresholds (CT) and normalized to GAPDH (ΔCT). The ΔCT values of the control were subtracted from each sample to gain the ΔΔCT values.

### 2.7. Evaluation of the Barrier Integrity of the BBB Models

To evaluate the barrier function in different BBB models, transendothelial electrical resistance (TEER) was measured by an EVOM resistance meter and Endohm chamber (World Precision Instruments, Sarasota, FL, USA). The resistance values of blank inserts (background resistance) were subtracted from values of inserts with cells. TEER data are shown as Ω × cm^2^. The permeability of sodium fluorescein (Na-F, MW: 376 Da) was also determined, and used as an index of paracellular transport. Cell culture inserts were transferred to 24-well plates containing 0.9 mL assay buffer (Dulbecco’s PBS (D-PBS) containing 0.9 mM CaCl_2_, 0.5 mM MgCl_2_, 4.5 g/L glucose, and 10 mM HEPES; pH 7.4). The luminal culture medium was replaced with 0.2 mL of assay buffer containing 10 μg/mL Na-F. Fifteen or 45 min after Na-F addition, the inserts were transferred to new wells containing the assay buffer. Na-F concentration in the collected samples was measured using a multiwell spectrophotometer (Wallac 1420 ARVO Multiabel Counter; Perkin Elmer, Waltham, MA, USA; excitation wavelength: 485 nm, emission wavelength: 535 nm). Transendothelial permeability coefficient (P_e_) was calculated as previously described [10].

### 2.8. Functional Assay for Efflux Transport

The functional activity of P-glycoprotein (P-gp) was determined by measuring cellular accumulation and bidirectional transport of rhodamine 123 (R123), a ligand of P-gp. After washing with the same assay buffer used for permeability studies, the cells were incubated in assay buffer containing 5 μM R123 for 1 h. They were then washed twice with ice-cold D-PBS, and lysed in 0.2 N NaOH. The R123 content was determined using a multiwell spectrofluorometer (excitation wavelength: 485 nm, emission wavelength: 535 nm). Cellular protein was measured using the BCA protein assay kit (Pierce, Rockford, IL, USA). Cyclosporin (10 μM, 15 min preincubation) was used as a reference P-glycoprotein inhibitor. For bidirectional transport assay, the flux of 5 μM R123 in assay buffer was measured for 1 h at 37 °C in the luminal-to-abluminal and in the opposite abluminal-to-luminal directions. After measuring the R123 content in samples collected from both compartments, P_e_ values were calculated.

### 2.9. Drug Permeability Experiments

The permeability of the same 10 drugs was measured in the apical-to-basolateral (A-B) and basolateral-to-apical (B-A) directions as described in our previous study [15]. All test compounds were dissolved in DMSO to yield a 10 mM solution, which was further diluted to 10 μM in HBSS-Hepes buffer (Hank’s Buffered Salt Solution containing 25 mM Hepes). The incubation time for each reference compound was as follows: 30 min for antipyrin, caffeine, verapamil, and indomethacin; 60 min for loperamide, quinidine, and digoxin; 120 min for cimetidine, atenolol, and vinblastine. The concentration of test compounds in the samples was determined by a Merck–Hitachi LaChrom HPLC with UV–VIS or a fluorescence detector (Merck, Darmstadt, Germany). Digoxin was measured on a Thermo LTQ XL linear ion trap mass spectrometer coupled with a Thermo Surveyor HPLC (San Jose, CA, USA). Apparent permeability coefficients (P_app_) were calculated using the following equation: Papp = (dQ/dT)/(A × C_0_), where dQ/dT is the cumulative amount in the receiver compartment versus time, A is the surface of the filter, and C_0_ is the initial concentration of the tracer in the luminal compartments.

### 2.10. Western Blotting

Protein samples from brain endothelial cells cultured in the presence or absence of pericytes and astrocytes were harvested by scraping in radioimmunoprecipitation assay buffer (RIPA; Santa Cruz Biotechnology, Dallas, TX, USA). An equal amount of protein in each sample was separated on a 4–15% Tris–Glycine eXtended gel (Bio-Rad, Hercules, CA, USA) and transferred onto Hybond™-P membranes (Amersham, Buckinghamshire, UK) which were incubated with anti-claudin-5, anti-occludin, and anti-ZO-1 antibodies at a dilution of 1:5000, anti-P-glycoprotein (GeneTex, Irvine, CA USA), anti-BCRP (Abnova, Taiwan), and anti-GLUT-1 (Abcam, Cambridge, UK) antibodies at a dilution of 1:2500, and anti-β-actin antibody (Sigma, loading control) at a dilution of 1:10,000 in 3% BSA in PBS. The source and catalogue number of antibodies is listed in Table S1. To visualize the immunoreactive bands, blots were incubated in Clarity Max Western ECL Substrate (Bio-rad). The bands were detected using a FluorChem SP Imaging System (Alpha Innotech Corp., San Leandro, CA, USA).

### 2.11. Statistical Analysis

All data are expressed as the means ± standard error of the mean (SEM). Statistical analysis of all data was determined using Student’s *t*-test for two group comparisons, and analysis of variance followed by the Tukey-Kramer method for comparison of multiple groups. A *p* value of less than 0.05 was considered statistically significant.

## 3. Results

### 3.1. Characterization of Monkey Brain Endothelial Cells

**Figure 1:**
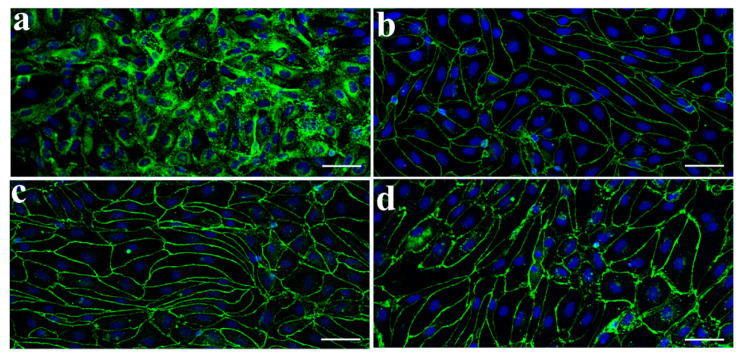
Characterization of primary monkey brain endothelial cells (MBECs) by immunofluorescence microscopy. MBECs express factor VIII-related antigen/von Willebrand factor (**a**). Tight junction protein ZO-1 (**b**), occludin (**c**), and claudin-5 (**d**) are expressed along the cell-cell border. Nuclear counterstain was stained with TO-PRO-3 (blue). Scale bar = 50 μm.

To characterize the morphology of primary MBEC cultures immunostaining was performed (Figure 1). The cells showed a spindle-shape morphology typical microvascular endothelial cultures and positive immunostaining for vWF, a marker for endothelial cells (Figure 1a). TJs formed between adjacent endothelial cells plays an important role in the restrictive property of the BBB. Immunostaining study revealed continuous and belt-like staining of the TJ proteins ZO-1 (Figure 1b), claudin-5 (Figure 1c), and occludin (Figure 1d) along the cell border in MBEC monolayers.

### 3.2. Expression of Transporters in Monkey Brain Endothelial Cells

**Figure 2:**
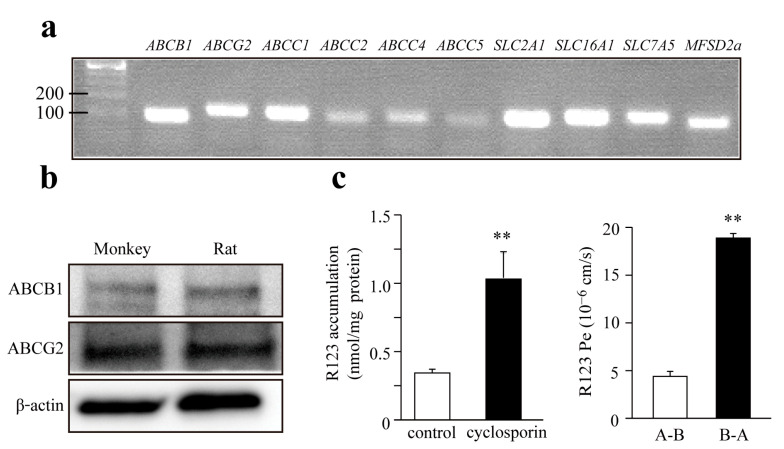
(**a**) Expression of transporters on monkey brain endothelial cells using PCR analysis; (**b**) Expression of efflux transporters ABCB1 and ABCG2 detected by western blot; (**c**) Functional analysis of ABCB1 using rhodamine 123 (R123) accumulation assay (**left**) and bi-directional transport of R123 (**right**). Accumulation of R123 was increased by ABCB1 inhibitor (cyclosporin; 10 μM). Transport of R123 in the basolateral to apical (B-A) was greater than apical to basolateral (A-B). Values presented are means ± SEM (n = 3–4, ** *p* < 0.01).

Brain capillary endothelial cells express several active efflux and influx transporters which contribute to the specific functions of the BBB. MBECs expressed mRNA for the efflux pumps P-gp (ABCB1), BCRP (ABCG2) and multidrug resistance-associated protein-1, 2, 4, and 5 (ABCC1, 2, 4, 5), as shown in Figure 2a. We also demonstrated the presence of nutrient transporters glucose transporter-1 (SLC2A1), monocarboxylate transporter-1 (SLC16A1), large neutral amino acids transporter-1 (SLC7A5), and the major facilitator superfamily domain containing protein-2A (MFSD2A) at gene level. The presence of the two most important ABC efflux pumps, P-gp and BCRP proteins, were verified by western blot both in monkey and rat primary BEC cultures (Figure 2b). The function of P-gp was further investigated by cellular uptake and bidirectional permeability studies using R123 (Figure 2c). A significant, more than three-fold increase, was measured in the intracellular accumulation of R123 in MBEC after treatment with cyclosporine A, an inhibitory substrate of P-gp (Figure 2c). P-gp expression is polarized in brain capillary endothelial cells, with a localization in the luminal membranes, facilitating the efflux of substrates from the brain side (basolateral) to the blood side (apical). In the bidirectional permeability assay, the transport of R123 from the brain side to the blood side was almost four-fold higher than in the opposite direction (Figure 2c) indicating a strong efflux pump activity.

### 3.3. Effect of Astrocytes on Barrier Function of Monkey Brain Endothelial Cells

**Figure 3:**
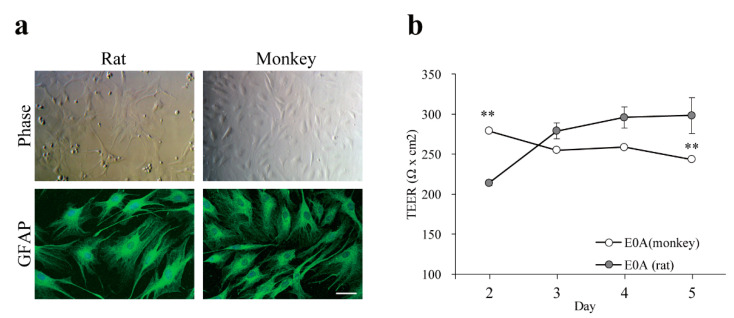
(**a**) Characterization of primary monkey astrocytes by immunofluorescence microscopy. Both monkey and rat astrocytes express glial fibrillary acidic protein (GFAP). Cell nuclei were counterstained with TO-PRO-3 (blue). Bar = 50 μm; (**b**) Barrier function of the co-culture BBB models with astrocytes. BECs from monkey were co-cultured with astrocytes from either monkey (E0A (monkey)) or rat (E0A (rat)). Barrier function was assessed by measuring TEER. Values presented are means ± SEM. (n = 3, ** *p* < 0.01). E00: monkey brain endothelial monolayers; E0A: co-culture of monkey brain endothelial cells and astrocytes (rat or monkey).

Primary monkey and rat astrocytes stained for glial fibrillary acidic protein showed a branched, stellate morphology (Figure 3). In a non-contact co-culture for 5 days the TEER values of monkey BECs were increased by rat primary astrocytes from neonatal animals, but not by monkey astrocytes isolated from adult animals (Figure 3b). For further experiments rat astrocytes and brain pericytes were used.

### 3.4. Barrier Integrity of Different BBB Models

**Figure 4:**
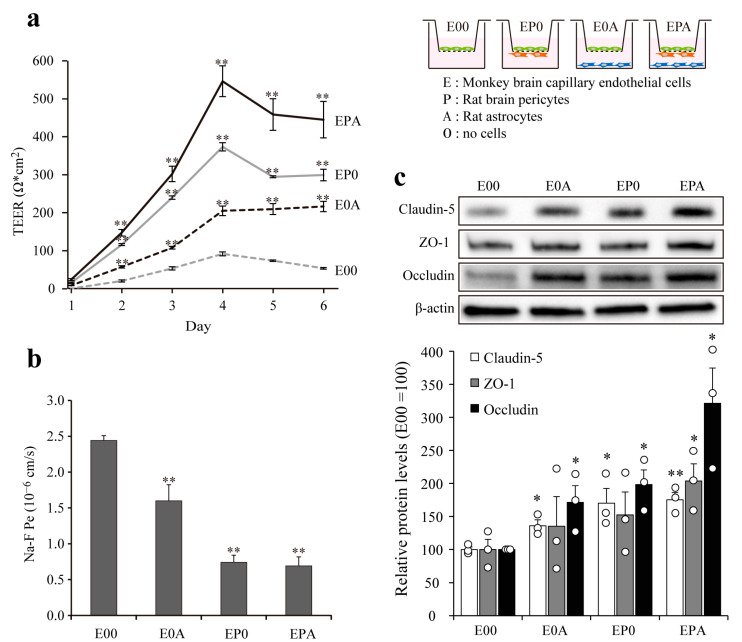
Comparison of the barrier properties of BBB mono- and co-culture models. Barrier function of different BBB models prepared from brain endothelial cells (E), astrocytes (A), and pericytes (P) were measured by (**a**) transendothelial electrical resistance (TEER, expressed as Ω × cm^2^) and (**b**) endothelial permeability coefficient for sodium fluorescein (Na–F P_e_, expressed in 10^−6^ cm/s). Data are presented as means ± SEM (n = 3, ** *p* < 0.01 vs. E00); (**c**) Expression of the tight junction proteins ZO-1, occludin, and claudin-5 in different BBB models detected by western blot. The relative level of the proteins was determined by densitometry. Data are presented as means ± SEM (n = 3, * *p* < 0.05, ** *p* < 0.01 vs. E00).

The investigated barrier functions of MBECs in mono- and different co-culture settings, TEER, and permeability for fluorescein were measured (Figure 4a,b).

Astrocytes and pericytes, which surround brain capillaries in vivo, contribute to the formation and maintenance of a functional BBB. Four types of in vitro BBB models were made from MBECs, primary rat astrocytes, and rat pericytes. The electrical resistance of MBEC monolayers (E00) stayed below 100 Ω × cm^2^ during the investigated 6-day period, while both rat primary astrocytes (E0A) and brain pericytes (EP0) significantly elevated the TEER values. The biggest effect on the paracellular tightness of MBECs was seen in the EPA co-culture model (545 ± 40 Ω × cm^2^ at day 4), when both cell types were present (Figure 4a). The permeability of the monolayer MBEC model (E00; 2.44 ± 0.04 × 10^−6^ cm/s) for fluorescein was the highest as compared to the co-culture models (Figure 4b). The lowest Na-F permeability was measured when brain pericytes were present in the co-culture models either alone (EP0; 0.74 ± 0.06 × 10^−6^ cm/s) or together with astrocytes (EPA; 0.69 ± 0.07 × 10^−6^ cm/s). The tightening effects of rat astrocytes and pericytes on the paracellular barrier of MBECs was demonstrated at the level of TJ proteins as well (Figure 4c). The expression of integral membrane TJ proteins claudin-5 and occludin were elevated in all three co-culture models as compared to the MBEC mono-cultures (E00), while the level of TJ linker protein ZO-1 was increased only in the EPA model (Figure 4c).

### 3.5. Expression of Transporters on Different BBB Models

**Figure 5:**
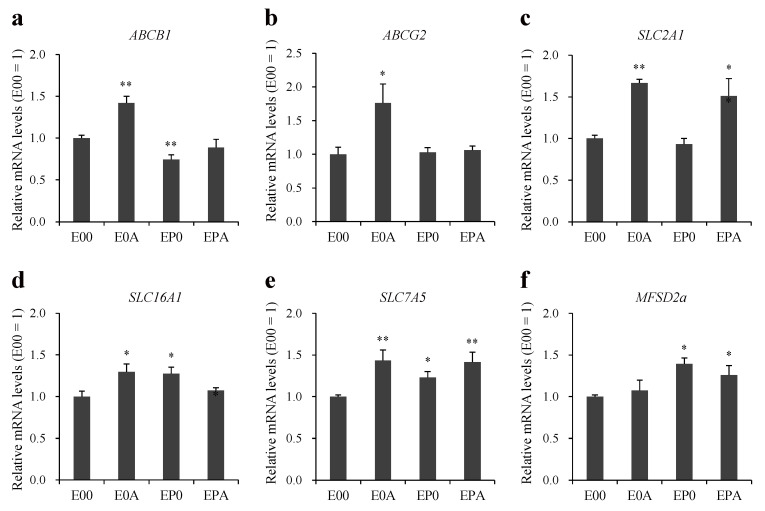
Expression of transporter genes (**a**) ABCB1; (**b**) ABCG2; (**c**) SLC2A1; (**d**) SLC16A1; (**e**) SLC7A5; and (**f**) MFSD2A in different BBB models. Expression of transporters were determined by quantitative PCR. All data are presented as means ± SEM (n = 4–9, * *p* < 0.05, ** *p* < 0.01 vs. E00).

The expression of key ABC and SLC transporter genes were determined in MBEC mono- and co-culture models (Figure 5). In the case of efflux transporters P-gp (ABCB1) and BCRP (ABCG2) an increase in the gene expression was observed by astrocytes (E0A), but not by other co-culture models (Figure 5a,b). Co-culture conditions did not change the expression of ABCC1, 2, 4, 5 genes in MBECs (Supplementary Figure S2).

Among the three SLC transporter genes, elevated expression of GLUT-1 was seen in MBECs in the E0A and EPA models (Figure 5c), while the level of MCT-1 was increased in the E0A and EP0 BBB models (Figure 5d) as compared to the monolayers cultured alone. The expression of LAT-1 gene was elevated in all three co-culture setups (Figure 5e). The gene expression of the lipid transporter MFSD2A was higher in MBECs (Figure 5f) when pericytes were present in the co-culture models (EP0 and EPA).

**Figure 6:**
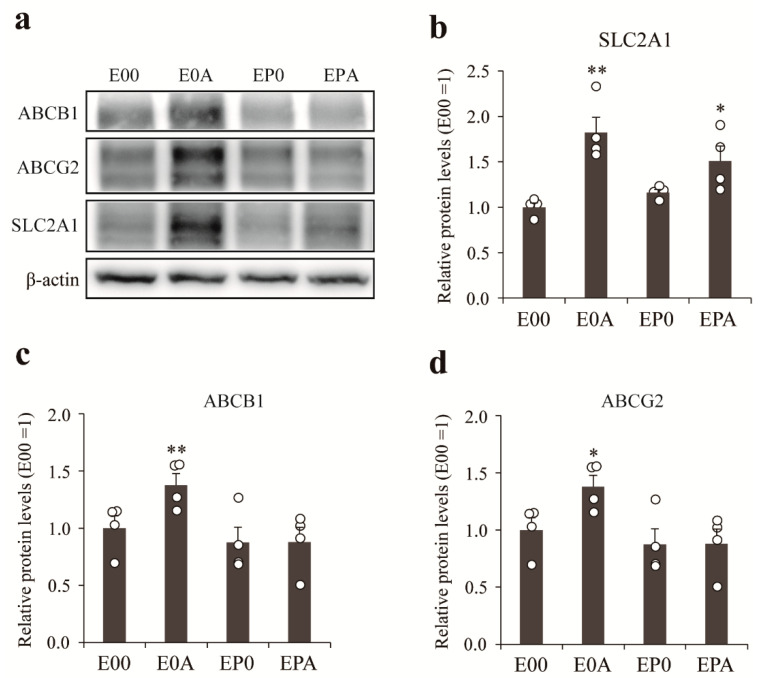
Expression of transporters in different BBB models were determined by western blot. (**a**) Representative image of the western blot. The bar graph reflects the quantitative analysis of the expression levels of SLC2A1 (**b**), ABCB1 (**c**), and ABCG2 (**d**) were determined in the different BBB models. All data are presented as means ± SEM (n = 4, * *p* < 0.05, ** *p* < 0.01 vs. E00).

The gene expression results were verified by western blot (Figure 6). The expression of GLUT-1 protein in MBECs was increased by co-culture with rat astrocytes either alone (E0A) or in the presence of rat pericytes (EPA; Figure 6b), while only astrocytes could elevate the expression of P-gp (Figure 6c) and BCRP (Figure 6d) as compared to mono-cultures of MBECs (E00).

### 3.6. Correlation between Permeability of Drugs Tested in Rat and Monkey BBB Co-Culture Models, and Mouse In Vivo Model

To investigate the differences in drug permeability between the monkey and rat in vitro co-culture (EPA) models, permeability tests were performed for 10 well known pharmacological compounds with different transport mechanisms (Table 2) used in our previous studies [8,15]. Caffeine, antipyrin, and indomethacin, lipophilic compounds with passive transcellular flux across the BBB, showed the highest P_app_ values for both the monkey and rat in vitro BBB models. The permeability values of the efflux pump ligands verapamil, loperamide and quinidine were also in the same range and decreasing order in the culture models.

The compounds showing the lowest P_app_ values were cimetidine, digoxin, vinblastine, and atenolol in both in vitro BBB models with slight differences in the ranking. The penetration of atenolol and digoxin was the two lowest in the rat in vitro and mouse in vivo models, while digoxin and vinblastine were the two compounds with the slowest permeability in the monkey model (Table 2).

**Figure 7:**
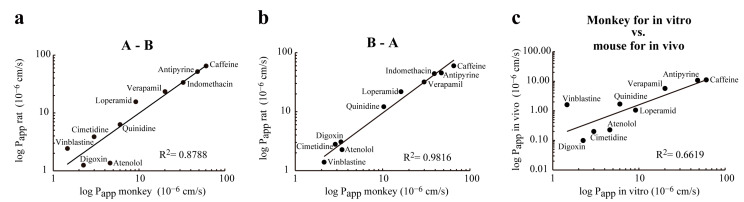
Correlation between permeability (Papp) data of drugs tested in in vitro rat BBB model, in vitro monkey BBB model, and mouse in vivo model. (**a**) Correlation between permeability of drugs in the apical to basolateral (A-B) tested at rat BBB model and monkey BBB model; (**b**) Correlation between permeability of drugs in the basolateral to apical (B-A) tested at rat BBB model and monkey BBB model; (**c**) Correlation between permeability of drugs tested at monkey BBB model (Papp in vitro) and the permeability of the same drugs measured in animal models (Papp in vivo).

When the P_app_ data measured in the A-B direction on the monkey BBB model were compared with the rat in vitro data, a high correlation (R^2^ = 0.88) was found (Figure 7a). The correlation between P_app_ data measured in the B-A direction was even higher (R^2^ = 0.98) when the rat and monkey co-culture BBB models were compared (Figure 7b). The lowest correlation (R^2^ = 0.66) was found between P_app_ values measured in the monkey in vitro BBB model and in vivo data obtained in mice (Figure 7c) indicating species differences in transporter activities.

### 3.7. Effect of TGF-β1 on Barrier Integrity in the Monkey Co-Culture BBB Model

**Figure 8:**
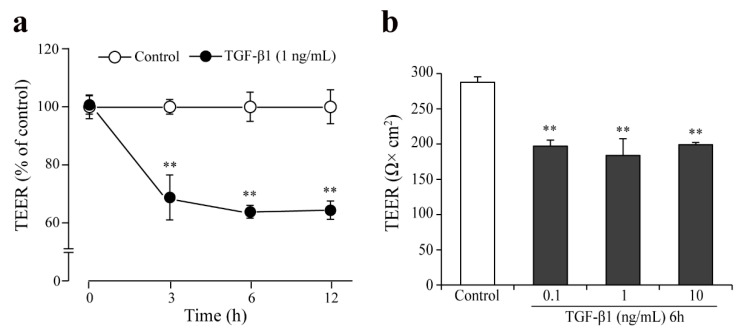
Effect of TGF-β1 on barrier integrity in the monkey co-culture BBB model (EPA). (**a**) Effect of TGF-β1 on the barrier integrity assessed by transendothelial electrical resistance (TEER) followed up to 12 h; (**b**) Effect of different concentrations of TGF-β1 on the TEER (6-h treatment). All data are presented as means ± SEM (n = 3, ** *p* < 0.01 vs. control).

To further prove the functionality of the monkey BBB co-culture model, the effects of the cytokine transforming growth factor β1 (TGF-β1) was tested on the barrier integrity (Figure 8). In the TGF-β1 (1 ng/mL) treated group a ~30% decrease in TEER values was measured at the 3, 6, and 12-h time points as compared to the medium treated group (Figure 8a). A similar effect on the barrier integrity was seen for 6-h TGF-β1 treatment in the concentration range of 0.1–10 ng/mL (Figure 8b).

## 4. Discussion

Cell-culture models are versatile tools to examine the molecular basis of the functions of brain endothelial cells in physiological and pathological conditions including drug transport [1,2,16]. The applicability of these systems in drug discovery depends on how well they can predict brain penetration in vivo [5]. While there are efforts to establish human cell-based BBB models for drug transport studies, no models fulfill all the validation criteria [17]. Species differences has been described in absolute protein expression levels of transporters in human, monkey, and mouse brain capillaries [12] confirming previous functional data. Among different species used in preclinical drug investigations, primates were found to be closest to humans in terms of BBB transport of P-gp ligands [18]. Culture models are important to replace, reduce, and refine experiments using animals, in line with the 3R principle. In the present study, a non-human primate co-culture BBB model consisting of primary monkey brain capillary endothelial cells, rat pericytes, and astrocytes was developed and characterized.

### 4.1. Characterization of the Primary Triple-Co-Culture Primate BBB Model

Primary cultures of cerebral endothelial cells, despite showing lower expression levels for some TJ, transporter, or receptor genes as compared to freshly isolated capillaries, still retain many of the in vivo properties, as it has been demonstrated for several species [3,6], including monkey cerebral endothelial cells [19]. The isolation and culture of monkey cerebral endothelial cells (*Macaca rhesus*) has been described 20 years ago [20,21]. This BBB model, the co-culture of monkey brain endothelial cells and monkey astrocytes, was characterized for immunological properties and used to study changes after simian immunodeficiency virus infection [22] or cytokine treatments [23]. In a recent study, the transcriptome of cerebral endothelial cells from *Macaca fascicularis* was compared between freshly isolated cells and cells kept in culture conditions [19]. The key elements of BBB functions, namely barrier integrity and control of molecular trafficking, were well preserved in MBECs in culture [19].

In our study, brain capillary endothelial cells isolated from *Macaca fascicularis* obtained by the puromycin selection method [14] expressed the most important transmembrane TJ proteins claudin-5, occludin and ZO-1, key solute and nutrient transporters and active efflux pumps ABCB1 and ABCG2 at the BBB, and showed a polarized transport of the P-gp ligand R123 (Figure 1 and Figure 2). The expression of the BBB transporters and TJ proteins are in concordance with previous works on monkey brain endothelial cultures [19,20], while the functional measurement of efflux pump activity is well supported by in vivo measurements [18].

To improve the paracellular barrier properties of the BBB model, MBECs were co-cultured with astrocytes isolated from adult monkey or rat neonatal brain cortex. Rat astrocytes increased the TEER of monkey brain endothelial cell layers in a time-dependent way, indicating tightened interendothelial junctions, in concordance with similar observations on rat, bovine, porcine and human cerebral endothelial cells [3]. Although the initial TEER of MBECs was high in the presence of monkey astrocytes, this effect did not last and the barrier inducing effect was lower than in the case of rat neonatal astroglia cultures (Figure 3). In a study comparing the gene expression of freshly isolated adult astrocytes with cultured adult and cultured neonatal astrocytes from rats, neonatal astrocytes expressed the highest levels for several growth factor genes, including basic fibroblast growth factor (FGF2) [24]. Since FGF2 [25] and other glia-derived growth factors tighten the barrier properties of BBB culture models [1], the higher expression of growth factors in neonatal rat astrocyte cultures as compared to adult monkey astrocyte cultures can explain the TEER differences. Brain pericytes also enhanced the TEER and decreased the permeability of MBECs layers, and the effect was even greater in the presence of both cell types (Figure 4), similarly to observations on rat brain endothelial cell models [10]. Astrocytes increased the expression of ABCB1 and ABCG2 at gene and protein levels, while the expression of GLUT1 was elevated in all co-culture models (Figure 5 and Figure 6). Our results show that astrocytes, pericytes, and their combination do not uniformly increase the level of different influx and efflux transporters, but a complex pattern of regulation is seen, which is in agreement with findings on porcine brain endothelial models [26].

The permeability of 10 selected pharmacons was tested on the monkey EPA model and compared to data measured on our rat EPA BBB model (Table 2, Figure 7). While a high correlation (R^2^ = 0.8788) was obtained when the permeability data from the monkey model were compared with the rat BBB model, the P_app_ values of atenolol and digoxin, ligands of human P-gp [27], were higher in the monkey model. The correlation of the in vitro data obtained on the monkey model with in vivo data obtained in mice was lower (R^2^ = 0.6619), partly due to these two ligands. Our observation indicates that for some substrates of P-glycoprotein rat brain capillary endothelial cells have higher P-gp activity than monkey endothelial cells. Indeed, lower expression of P-glycoprotein was found in monkey and human brain capillaries than in mouse brain capillaries by a quantitative targeted absolute proteomics method [12]. In addition, a PET study demonstrated that penetration of P-gp substrates, such as [^11^C]GR205171, was greater into human and monkey brain than into rodent brain [18]. The protein expression levels of P-gp, BCRP, and MRP4 efflux transporters were similar in isolated human and monkey brain capillaries, only OATP1A2, expressed at low level in monkey brain capillaries, was under detection limit in human samples [12]. The present findings agree with these observations and suggest that the triple co-culture model using monkey endothelial cells can be a useful model to predict the brain penetration of P-gp ligands.

To test the applicability of the model to study pathological conditions, we have treated the EPA BBB model with TGF-β1 and found that this cytokine significantly decreases the TEER of the model, indicating paracellular opening (Figure 8). Similar effects were seen in bovine brain endothelial cells [1]. Our finding, together with observations on cytokine treatments and virus infection [22,23], indicate that the monkey model could be also used for the study of inflammatory conditions related to the BBB.

We should also note, that the limitations of primary cell based primate co-culture BBB models include that they are more expensive than cell line models, the isolation process is lengthy and need technical expertise, and tissue availability may depend on geographical location, ethical regulations, and permissions.

### 4.2. Application of the Primary Triple-Co-Culture Primate BBB Model

Since the preliminary publications about our model [28,29] a ready to use in vitro BBB model made of primary cultures of monkey (*Macaca* *fascicularis*) brain capillary endothelial cells, rat brain pericytes and astrocytes have been commercially available at PharmaCo-Cell Co. Ltd. (Nagasaki, Japan) [30]. This primary triple co-culture non-human primate BBB model has been widely used for various biomedical and pharmaceutical applications, and some results have been already published during recent years [31,32,33,34,35,36,37,38,39].

Our primate BBB model has been successfully applied for testing the BBB penetration of novel nonpeptidic human immunodeficiency virus type 1 (HIV-1) protease inhibitors and selecting drug candidates with promising brain-targeting properties [31,32,34,36]. Permeability data across the monkey BBB model were provided not only for novel nonpeptidic HIV-1 protease inhibitor compounds GRL-083-13, GRL-084-13, and GRL-087-13 (mean P_app_ values in the 55.5 to 72.0 × 10^−6^ cm/s range), but also for known conventional anti-HIV-1 drugs, including protease inhibitors saquinavir, amprenavir, lopinavir, and darunavir (mean P_app_ values in the 4.9 to 15.4 × 10^−6^ cm/s range); nucleoside reverse transcriptase inhibitors 3′-azido-2′,3′-dioxythymidine, lamivudine, tenofovir disoproxil fumarate and emtricitabine (mean P_app_ values in the 9.2 to 23.2 × 10^−6^ cm/s range); nonnucleoside reverse transcriptase inhibitors nevirapine and efavirenz (mean P_app_ values are 32.0 and 14.4 × 10^−6^ cm/s, respectively); integrase inhibitors raltegravir and elvitegravir (mean P_app_ values are 10.2 and 18.6 × 10^−6^ cm/s, respectively); and C-C chemokine receptor type 5 (CCR5) inhibitor maraviroc (mean P_app_ value is 16.0 × 10^−6^ cm/s) [36]. Permeability data for reference drug with high brain penetration, caffeine (P_app_ = 71.7 ± 3.2 × 10^−6^ cm/s), and passive paracellular marker sucrose (P_app_ = 1.0 ± 0.3 × 10^−6^ cm/s) measured in this experiment [36] confirm the adequacy of the model system for testing in vitro penetration of drug candidates targeted to the central nervous system (CNS) [3]. These data are also in accordance with our data set shown in Table 2 and Figure 7.

The model was also used to test the BBB permeability of a natural compound, myricetin, a polyphenolic flavonoid, beneficial in the prevention of amyloid β_1-42_ peptide-induced neuronal cell damage, and a P_app_ value of 2.5 × 10^−6^ cm/s was measured [38]. Furthermore, the functional expression the drug transporter SLC35F2 (solute carrier family 35 member F2) was demonstrated using our in vitro BBB model [39]. In this study, the quality of the monkey BBB kits was validated by low paracellular permeability (TEER value of 330 Ω × cm^2^ and low Lucifer yellow transport) and active efflux transport of P-glycoprotein substrate quinidine [39]. Cultured monkey brain endothelial cells expressed SLC35F2 transporter mRNA and protein, and transport experiments confirmed famotidine-inhibitable BBB permeability and intraendothelial accumulation of YM155 (sepantronium bromide), an SLC35F2 substrate [39].

This in vitro BBB model was also applied to prove that oxytocin is transported to the CNS by receptors for advanced glycation end-product (RAGE) [37]. Dose-dependent blood-to-brain direction transport of oxytocin was reduced by approximately 70% in monkey brain endothelial cell monolayers when RAGE was silenced by shRNA, while TEER values of 800 Ω × cm^2^ were unchanged in knockout cells [37].

Four types of monoclonal antibodies specific to the extracellular domains of human claudin-5 (anti-CLDN-5 mAb) dose-dependently attenuated TEER and increased the BBB permeability for Na-F and fluorescein isothiocyanate-labeled dextran (MW: 4 kDa) in our triple culture monkey model [35]. The efficacy of R9, the most potent anti-CLDN-5 mAb selected on the in vitro primate model, has been recently confirmed in vivo, and 3 mg/kg intravenous dose resulted in increased BBB permeability to Na-F in male cynomolgous monkeys (*Macaca fascicularis*) indicating that claudin-5 may be a potential target to increase CNS drug delivery in humans [40]

In addition, an in vitro cancer metastasis model was established using our monkey BBB model [33]. Extracellular vesicles derived from human breast cancer cells deteriorated BBB properties (reduced TEER and increased P_app_ for sodium fluorescein) through the change in actin dynamics in vitro and promoted metastatic transport of cancer cells to the abluminal space [33]. It was proved that microRNA-181c promoted the dysfunction of BBB through delocalization of actin fiber via the downregulation of 3-phosphoinositide-dependent protein kinase-1 (PDPK1) [33]. The applicability of the presented non-human primate model in a wide variety of biomedical and drug discovery experiments and the consistency of permeability data accumulated at various laboratories strengthen the claim that the ready-to-use monkey BBB kits are suitable for academic research and the pharmaceutical industry.

## 5. Conclusions

We have developed and characterized a non-human primate co-culture BBB model using primary monkey brain endothelial cells, rat brain pericytes, and astrocytes. Monkey brain endothelial cells in the presence of both pericytes and astrocytes expressed enhanced barrier properties and elevated levels of key BBB influx and efflux transporters. A good correlation was found between the endothelial permeability coefficients of 10 well known drugs in the monkey and rat BBB culture models. A lower correlation was found between the permeability values of the drug set when the monkey culture model was compared to mouse in vivo data, indicating transporter differences between species. The characterized monkey BBB model has been applied in several biomedical and drug discovery studies and can be a valuable tool for basic and applied pharmaceutical research.

## Figures and Tables

**Table 1:** Primers used for PCR analysis.

Gene	Primer Sequence	Size (bp)
ABCB1 (P-gp)	F: GGCCTAAAGCCGAACACATTG R: CTGAAGCACTGGGATGTCCA	90
ABCG2 (BCRP)	F: GCCACGGAGATCATAGAGCC R: TCACCCCCGGAAAGTTGATG	125
ABCC1 (MRP-1)	F: CAAGGGATTGCCGTGTTTGG R: AAGAAGCTCATGGGTGACCG	116
ABCC2 (MRP-2)	F: GCACAAGCAACTGCTCAACA R: CCGTGGAAATATCACCGGCA	104
ABCC4 (MRP-4)	F: TCGCAATACCCTTGGTTCCC R: CACTGGGCTCCGAGTTGTAG	110
ABCC5 (MRP-5)	F: CTTTGTCAAGGGCACACTGC R: CCTGTGGGGGTTGTGTCAAA	102
SLC2A1 (GLUT1)	F: GAACTCTTCAGCCAGGGTCC R: GGACCACATAGTTGCTCCAC	116
SLC16A1 (MCT1)	F: ACAAGTAAACGAGGCAGCGA R: ACAAATATCGTTATAAGCGCGGA	123
SLC7A5 (LAT1)	F: CGTGAACTGCTACAGCGTGA R: TTGGACACATCACCCTTCCC	126
MFSD2a	F: GCCCAGGTGAAGAAAGAACC R: CACAGCCTGTCACCTGGTAG	103
GAPDH	F: CTCAAGATCGTCAGCAACGC R: TCTTCTGGGTGGCAGTGATG	130

**Table 2:** BBB permeability parameters measured in monkey and rat in vitro models and in mouse in vivo.

Compound	Primary Transport Mechanism	Monkey BBB			Rat BBB *			Mouse In Vivo *
		P_app_ A-B (×10^−6^ cm/s)	P_app_ B-A (×10^−6^ cm/s)	EffluxRatio	P_app_ A-B (×10^−6^ cm/s)	P_app_ B-A (×10^−6^ cm/s)	EffluxRatio	P_app_ (×10^−6^ cm/s)
Caffeine	PT/CMT	60.53	65.25	1.08	64.95	59.91	0.92	11.11
Antipyrin	PT	48.06	47.07	0.98	51.78	45.38	0.88	10.75
Indomethacin	PT	32.70	39.11	1.20	33.63	44.07	1.31	0.13
Verapamil	PT/E	20.10	29.86	1.49	23.38	31.82	1.38	5.69
Loperamide	PT/E	9.16	16.26	1.78	15.57	21.76	1.47	1.05
Quinidine	PT/E	6.01	10.33	1.74	6.28	12.11	1.93	1.69
Cimetidine	E	3.00	2.88	0.97	3.86	2.80	0.75	0.20
Digoxin	E	2.25	3.34	1.49	1.25	3.07	2.47	0.10
Vinblastine	E	1.47	2.14	1.46	2.42	1.39	0.69	1.43
Atenolol	PP/E	4.60	3.43	0.83	1.36	2.26	1.71	0.23

* Data are obtained from [15]. Abbreviations: CMT, carrier mediated transport; E, efflux transport; PP, passive paracellular; PT, passive transcellular; P_app_, apparent permeability coefficient; P_app_ A-B, P_app_ measured in apical to basal (luminal to abluminal) direction, P_app_ B-A, P_app_ measured in basal to apical (abluminal to luminal) direction.

## Data Availability

The data presented in this study are available on request from the corresponding author.

## Supplementary Materials

The following are available online at https://www.mdpi.com/article/10.3390/pharmaceutics13091484/s1, Supplementary Method: Culture of rat brain capillary pericytes, Culture of rat cerebral astrocytes, Table S1: Antibodies used for immunohistochemistry and western blot, Figure S1: Expression of ABCC transporters in different BBB models. Figure S2. Expression of ABCC transporter genes in different BBB models determined by quantitative PCR. (a) Expression of ABCC1 transporter; (b) Expression of ABCC2 transporter; (c) Expression of ABCC4 transporter; (d) Expression of ABCC4 transporter. All data are presented as means ± SEM (n = 5–7, * *p* < 0.05 vs. E00). E00: monkey brain endothelial monolayers; E0A: co-culture of monkey brain endothelial cells and rat astrocytes; EP0: co-culture of monkey brain endothelial cells and rat brain pericytes; EPA: co-culture of monkey brain endothelial cells and rat astrocytes and rat brain pericytes.
