# Supplementary material for: Characterization of a Primate Blood-Brain Barrier Co-Culture Model Prepared from Primary Brain Endothelial Cells, Pericytes and Astrocytes
Source: Pharmaceutics. 2021 Sep 16;13(9):1484. doi: 10.3390/pharmaceutics13091484 (PMC8470770; doi:10.3390/pharmaceutics13091484)
Supplement: Supplementary file 1 [file pharmaceutics-13-01484-s001.zip › pharmaceutics-1338573-supplementary.pdf]

SUPPLEMENTARY MATERIAL

Characterization of a primate blood-brain barrier co-culture model prepared from primary brain endothelial cells, pericytes and astrocytes

Daisuke Watanabe [1,2], Shinsuke Nakagawa [3], Yoichi Morofuji [4], Andrea E. Tóth[5#], Monika Vastag [6], Jun Aruga [1], Masami Niwa [2] and Mária A. Deli [5,*]

[1]Department of Medical Pharmacology, Nagasaki University Graduate School of Biomedical Sciences, Nagasaki 852-Japan; [2]BBB Laboratory, PharmaCo-Cell Co. Ltd., Nagasaki, Japan; [3]Department of Pharmaceutical Care and Health Sciences, Faculty of Pharmaceutical Sciences, Fukuoka University, Fukuoka Japan; [4]Department of Neurosurgery, Nagasaki University Graduate School of Biomedical Sciences, Nagasaki, Japan; [5]Institute of Biophysics, Biological Research Centre, Szeged, Hungary; [6]Division of Pharmacology and Drug Safety Research, Gedeon Richter Plc., Budapest, Hungary; *Correspondence: deli.maria@brc.hu

---

Supplementary Method

1. Culture of rat brain capillary pericytes

Pure cultures of rat cerebral pericytes were obtained from primary isolated brain microvessel fragments that contained pericytes and endothelial cells, so the protocol was adapted from the described technique in the section for brain capillary endothelial cells. Pericytes survival and proliferation were favored by selective culture conditions: shortening the second enzyme digestion to 15 min, using uncoated dishes, and DMEM supplemented with 10% fetal bovine serum (FBS) and antibiotics. Isolated microvessel fragments from two rat brains were seeded into one 100-mm culture dish coated with 0.1 mg/mL type IV collagen (Sigma). Endothelial cells and pericytes migrated out of the vessel fragments the next day after isolation. After the mixed cultures were maintained in 10% FBS/DMEM for 5-7 days, the cells were passaged by a brief treatment with trypsin (0.05% wt/vol) and EDTA (0.02% wt/vol) solution, and plated to uncoated 100-mm dishes. The culture medium was changed every 3 days. Pure cultures of rat cerebral pericytes were obtained 4-6 days later. The total length of the culture of brain pericytes lasted ~2 weeks. Pericytes were used at passage 2-3. Pericytes were characterized by their large size and branched morphology, positive immunostaining for α-smooth muscle actin and NG2 chondroitin sulfate proteoglycan, and the absence of von Willebrand factor and glial fibrillary acidic protein (GFAP) staining as shown in our previous publication (Nakagawa et al., 2009).

2. Culture of rat cerebral astrocytes

Rat cerebral astrocytes were obtained from neonatal Wistar rats. Meninges were removed and cortical pieces were mechanically dissociated in DMEM. Dissociated cells were passed through a 70-µm nylon mesh (BD Falcon) and seeded into cell culture flasks. In

order to obtain astrocytes as purely as possible, flasks with confluent cultures were shaken strongly by hand to remove microglia. This procedure was performed three times during a two-week culture period. The purity of astrocytes was verified by immunostaining for GFAP (Figure 3a), and the cells were used at passage 2-3. Pericytes and astrocytes were frozen in cryo-medium Cellbanker (BCL-1, Zenoaq, Koriyama, Japan), and stored at -80 °C until use.

Reference

Nakagawa, S.; Deli, M.A.; Kawaguchi, H.; Shimizudani, T.; Shimono, T.; Kittel, Á.; Tanaka, K.; Niwa, M. A new blood-brain barrier model using primary rat brain endothelial cells, pericytes and astrocytes. Neurochem Int. 2009, 54, 253-263. doi:10.1016/j.neuint.2008.12.002.

Table S1. Antibodies used for immunohistochemistry and western blot.

Antibody	Company	Catalogue number
anti-claudin-5	Invitrogen	35-2500
anti-occludin	Invitrogen	33-1500
anti-ZO1	Invitrogen	33-9100
anti-vWF	Sigma	F3520
anti- GFAP	Progen Scientific	61011
anti-β-actin	Sigma	A5441
anti-P-gp	GeneTex	GTX23364
anti-BCRP	Abnova	H00009429-B01P
anti-GLUT1	Abcam	ab15309
A488 conjugated donkey anti-rabbit	Invitrogen	A-21206
A488 conjugated donkey anti-mouse	Invitrogen	A-21202
Anti-mouse IgG, HRP-linked	Cell Signaling Technology	7076
Anti-rabbit IgG, HRP-linked	Santa Cruz Biotechnology	sc-2357

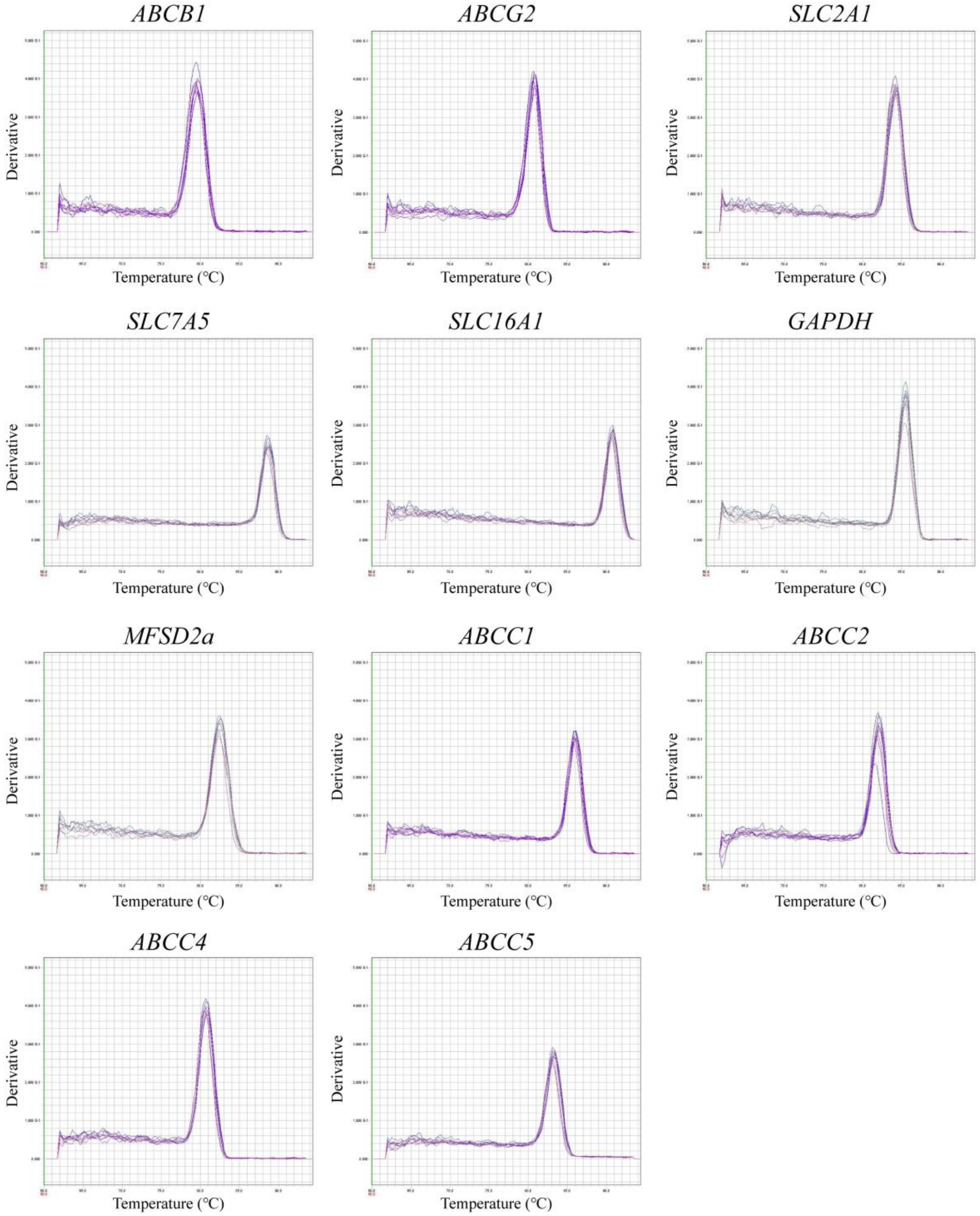

Figure S1. Melting curve images for the primers used for the PCR analysis listed in Table 1.

.

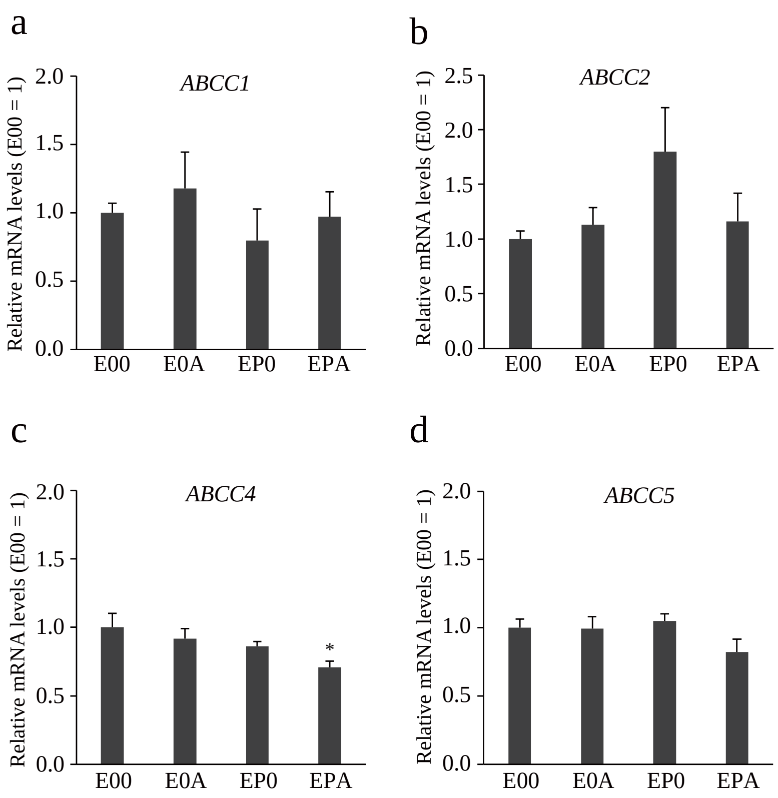

Figure S2. Expression of ABCC transporter genes in different BBB models determined by quantitative PCR. a) Expression of ABCC1 transporter; b) Expression of ABCC2 transporter; c) Expression of ABCC4 transporter; d) Expression of ABCC4 transporter. All data are presented as means ± SEM (n = 5-7, *$p < 0.05$ vs. E00). E00: monkey brain endothelial monolayers; E0A: co-culture of monkey brain endothelial cells and rat astrocytes; EP0: co-culture of monkey brain endothelial cells and rat brain pericytes; EPA: co-culture of monkey brain endothelial cells and rat astrocytes and rat brain pericytes.
